# Detection of polymorphism within leptin gene in Egyptian river buffalo and predict its effects on different molecular levels

**DOI:** 10.1186/s43141-020-0020-5

**Published:** 2020-02-10

**Authors:** Karima F. Mahrous, Mohamad M. Aboelenin, Mohamed A. Rashed, Mahmoud A. Sallam, Hossam E. Rushdi

**Affiliations:** 1 Cell Biology Department, National Research Centre, Giza, Egypt; 2 Department of Genetics, Faculty of Agriculture, Ain Shams University, Cairo, Egypt; 3 Department of Animal Production, Faculty of Agriculture, Cairo University, Giza, Egypt

**Keywords:** Egyptian river buffaloes, Leptin, PCR-RFLP, SNP

## Abstract

**Background:**

Leptin (LEP) regulates the glucose homeostasis directly and centrally by the regulation of the insulin levels or indirectly by alternation of the levels of the other glucose metabolism regulator hormones. The present investigation studied the polymorphism in LEP gene which is related to fertility in 81 female Egyptian river buffalo.

**Results:**

The PCR-RFLP pattern of the gene using the restriction enzyme Eco91I showed that all the animals had monomorphic pattern in the studied gene which consists of CC. A 511-bp fragment from LEP gene was amplified and sequenced. The homology between the amplified LEP gene fragment in buffalo and cattle, sheep, goat, human, and mouse on the nucleotides sequence level was 99, 97, 97, 87, and 79%, respectively, and on the translated amino acids sequence level was 100, 98, 98, 85, and 82%, respectively. Several SNPs were detected; among them, the T27C SNP disrupted an intronic splicing silencer. The A114G, A310G, G263A, and G379A SNPs disrupt exonic splicing enhancers, and the last two SNPs create new exonic splicing enhancers. The A114G, C163A, A211G, G288A, A310G, A322G, G330C, C348T, T360C, and G379A SNPs cause S71G, T87 N, N103S, E129K, E136G, Y140C, E143Q, R149W, S153P, and R159Q amino acids mutations. N103S, E129K, E136G, Y140C, E143Q, and S153P were classified as deleterious mutations. Y140, E143, N103, and R149 were the most conserved among the mutated amino acids. S71G only increased the stability of the leptin protein while the remaining mutations decreased it.

**Conclusion:**

Four SNPs were revealed among the tested animals. Twenty-one SNPs were found between the sequenced amplicon and the buffalo records in the Genbank. Some SNPs were predicted to have several effects on different biological processes like mRNA splicing, protein stability, and the gene functions.

## Background

Leptin (LEP) is a hormone mainly synthesized in the white adipose tissue in addition to other tissues. The premature and inactive form of leptin protein consists of 167 amino acids, but their mature functional polypeptides consist of 146 amino acids [1]. Leptin has important role in the energy and glucose homeostasis and plasma glucose levels [2]. Vallinoto et al. [3] revealed that the river buffalo and cattle have the same basic structure of leptin gene which consists of 3 exons separated with 2 introns, the start and stop codons are located in exon 2 and exon 3, respectively, but exon 3 is untranslatable. The authors identified 1 microsatellite and 3 SNPs (single nucleotide polymorphism) in the buffalo leptin gene. They recognized the chromosomal location of the river buffalo leptin gene on BBU8q32.

Liefers et al. [4] used polymerase chain reaction-restriction fragment length polymorphism (PCR-RFLP) to genotype 323 Holstein-Friesian heifers based on A59V SNP mutation in leptin protein due to C to T SNP placed in the position 95 from the start of LEP-exon 3 which change an amino acid in position 59 of leptin protein from alanine to valine. They took blood samples from each animal starting from 30 days before to 80 days after parturition, and the leptin concentrations were analyzed in each sample. The authors found that the animals with TT genotype had high leptin concentration during the last 30 days of pregnancy compared to CC and CT genotypes. Yazdani et al. [5] investigated the effect of A59V variant in leptin protein in 255 Iranian Holstein cows using RFLP-*HphI*, and they indicated that the AA genotype had pregnancy length that is significantly longer than AB and BB genotypes, and also, Clempson et al. [6] studied A59V replacement within leptin protein in 509 Holstein Friesian heifers and showed that the heifers with CC genotype were younger at the first service and first calving ages. Komisarek and Antkowiak [7] screened 219 Jersey cows using PCR-RFLP method by restriction enzyme *Eco91I* (RFLP-*Eco91I*) to identify the relationship between fertility and A59V SNP. TT genotype had shorter time of days open and calving interval in addition to lower number of inseminations per conception than CC and CT genotypes.

Orrú et al. [8] discovered 1 SNP in exon 2, 5 SNPs in exon 3, and 8 intronic SNPs by analysis; the sequence of 2 fragments represents exons 2 and 3 and part of intron 1 that covers all the leptin gene coding sequence in 32 Italian River Buffalo and 2 Egyptian river buffalo, while Seong and Kong [9] investigated the sequence of leptin gene-exon 3 in 20 American Murrah Buffalo and 350 Bulgarian Murrah Buffalo and found 3 SNPs between these populations.

Kale et al. [10] discovered 2 SNPs within the exon 3 of leptin gene in 65 Murrah, 10 Surti, and 10 Bhadawari breeds of buffaloes using the single-stranded conformation polymorphism (SSCP) technique and the sequencing of the amplified region. However, Datta et al. [11] analyzed and studied the sequence of intron 1, exons 2, intron 2, and exons 3 within leptin gene among Murrah buffaloes and indicated variations in 8 positions compared to cattle. Di Gregorio et al. [12] explored the sequence of a region within leptin gene covers a part of intron 1, full exon 2, and part of Exon 3 and represents the complete coding sequence for the 167 amino acids of the premature leptin in the water buffalo, goat, cattle, and sheep. They indicated that the sequence of the leptin gene in goat and sheep is more homologous than water buffalo and cattle.

The present study aimed to investigate the genetic polymorphisms in leptin gene (LEP) within Egyptian buffalo females which could be used as molecular markers in marker-assisted selection (MAS)-based breeding programs to improve the fertility of Egyptian buffalo females.

## Methods

This investigation was carried out in the Cell Biology Department, National Research Centre, Giza, Egypt.

### Sample collection and DNA extraction

A total of 81 blood samples were collected from healthy and unrelated Egyptian river buffalo females. Thirty blood samples were collected from the farm of Cattle Information System/Egypt (CISE), and 51 blood samples were collected from the Agricultural Experiments Station (AES), Faculty of Agriculture, Giza, Egypt. Ten milliliters of blood samples were collected in sterile 15 ml tubes containing 0.5 ml of 0.5 M EDTA solution (pH 8.0). Genomic DNA was extracted from the whole blood samples according to the method described by Miller et al. [13] with minor modifications. The DNA concentration was determined using Nano Drop1000 thermo scientific spectrophotometer and then diluted to the working final concentration of 50 ng/μl.

### Animal genotyping and leptin amplicon sequencing

A DNA fragment which is a part of LEP gene was amplified using forward (5′TGCCCTCTCTCCCACTGA3′) and reverse (5′CTGGTGAGGATCTGTTGGTAGGTC 3′) primer pair which were designed using Primer3 online software (http://primer3.ut.ee/; [14]) based on the sequence of GenBank record HE605297.1. Polymerase chain reaction (PCR) was performed in 25 μl of reaction volume, which included 50 ng of genomic DNA, 50 ng of each primer, 200 μM of each dNTP, 2.5 μl of 10× PCR buffer, and 0·5 U of Taq DNA polymerase (Promega, Madison, WI, USA). Amplification was carried out in a thermocycler which was programmed as follows: an initial start separation cycle at 94 °C for 2 min, 35 cycles including a denaturation step at 94 °C for 30 s, an annealing step at 60 °C for 30 s, a polymerization step at 72 °C for 45 s, and a final extension cycle at 72 °C for 10 min. The PCR products were screened by electrophoresis on a 2% agarose gel in 0.5× of TBE buffer which was stained with ethidium bromide and visualized with an UV transilluminator. PCR product was digested by *Eco91I* restriction enzyme (Thermo Scientific, Dreieich, Germany) at 37 °C for 30 min according to the procedure provided by the manufacturer. Digested products were separated by electrophoresis on a 2% agarose gel in 0.5× of TBE buffer which was stained with ethidium bromide and visualized with an UV transilluminator [15, 16].

### Purification and sequencing of PCR products

A longer segment from leptin gene was amplified using the same forward primer (5′TGCCCTCTCTCCCACTGA3′) in addition to new reverse primer (5′CCGCTGGCCTGCATAAAG 3′) primer pair. The PCR products of selected samples were purified using GeneJET Gel Elution Kit (Thermo Scientific, Dreieich, Germany). The purified PCR products were sequenced using an automated sequencing service (Macrogen, Korea).

### Data analysis

Alignment of the sequences was performed with the GenBank database BLAST tool (https://blast.ncbi.nlm.nih.gov/Blast.cgi) to identify the homology between the amplified LEP fragment and the orthologues sequences on the GeneBank database. The splicing site strength was predicted using SplicePort [17], and the effects of discovered SNPs and RNA cis-regulatory elements were investigated using RegRNA 2.0 [18] online software. To predict the effects of the non-synonymous SNPs on protein functions, the consensus classifier software PredictSNP was used which combined the results of MAPP, PhD-SNP, PolyPhen-1, PolyPhen-2, SIFT, and SNAP software [19]. The 3D tertiary structure of the proteins was predicted using the I-TASSER server [20], and the effect of the detected mutation on the protein stability was calculated by the INPS3D server [21]. The conservation score of the amino acids within the target proteins was computed using the ConSurf server [22].

## Results

**Fig. 1:**
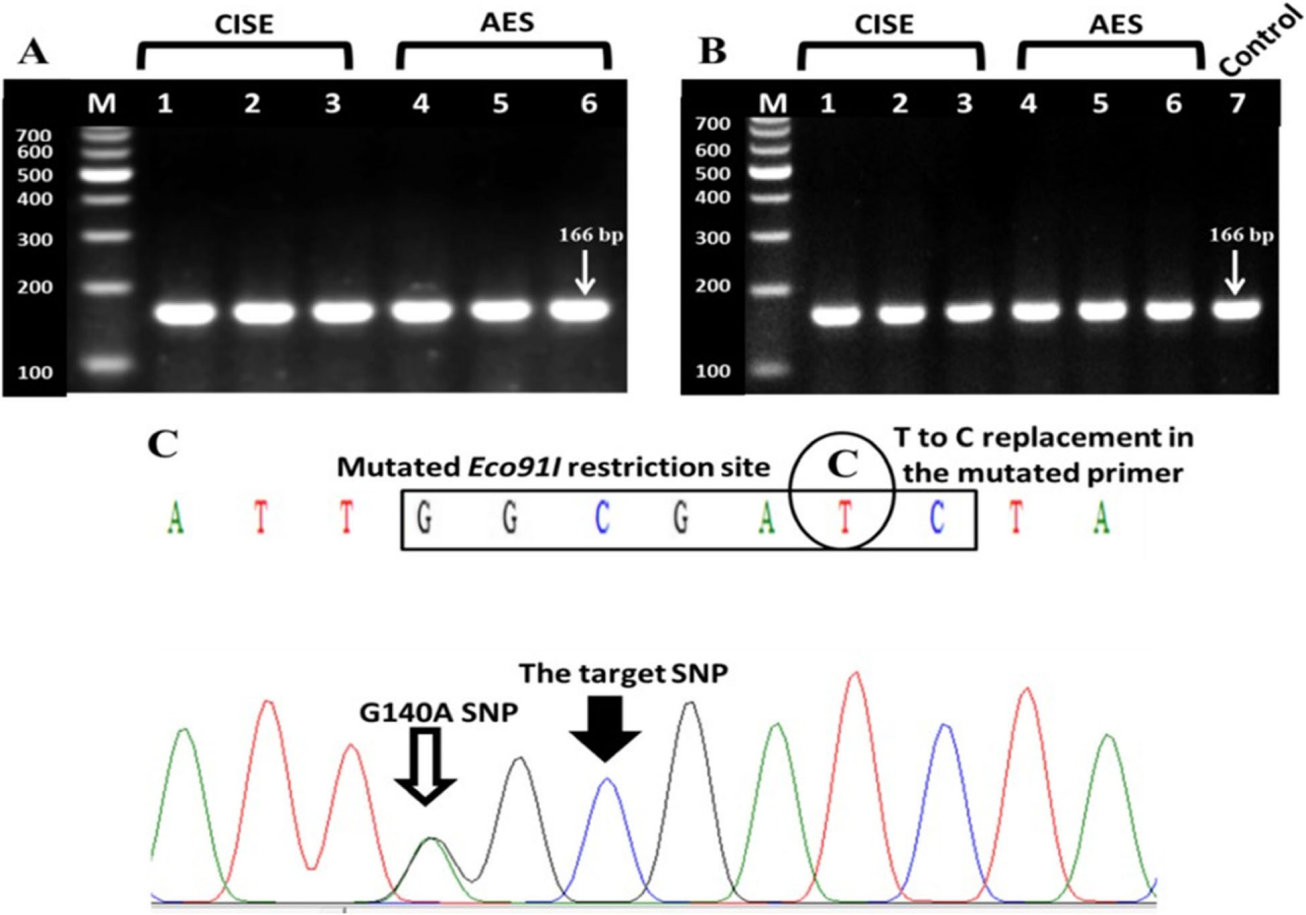
The PCR product, PCR-RFLP pattern, and sequencing result of the amplified LEP gene fragment. **a** The PCR product of LEP fragment. **b** The PCR-RFLP pattern of the LEP gene amplicon. **c** A part of LEP fragment sequencing chromatogram. CISE: Cattle Information System/Egypt. AES: Agricultural Experiments Station. M: 100 bp molecular marker

A fragment from the buffalo LEP gene (166 bp) was amplified by PCR using a mutated forward primer (Fig. 1a, c), and the amplicons were digested by *Eco91I*. PCR-RFLP pattern showed that all the buffalos had a single undigested band with molecular weight of 166 bp which refers to CC genotype (Fig. 1b). PCR-RFLP results were confirmed by direct sequencing of 511 bp amplified fragment within LEP gene (using the same reverse primer and a new forward primer). The sequencing results (Fig. 1c) show that the C allele (which codes for the amino acid Ala) was found in the target site. Allele C mutates the *Eco91I* enzyme restriction site (GG**T**NACC, allele T) to an unrestrictable site (GG**C**NACC, allele C).

### Sequencing of the amplified sequence from LEP gene

**Fig. 2:**
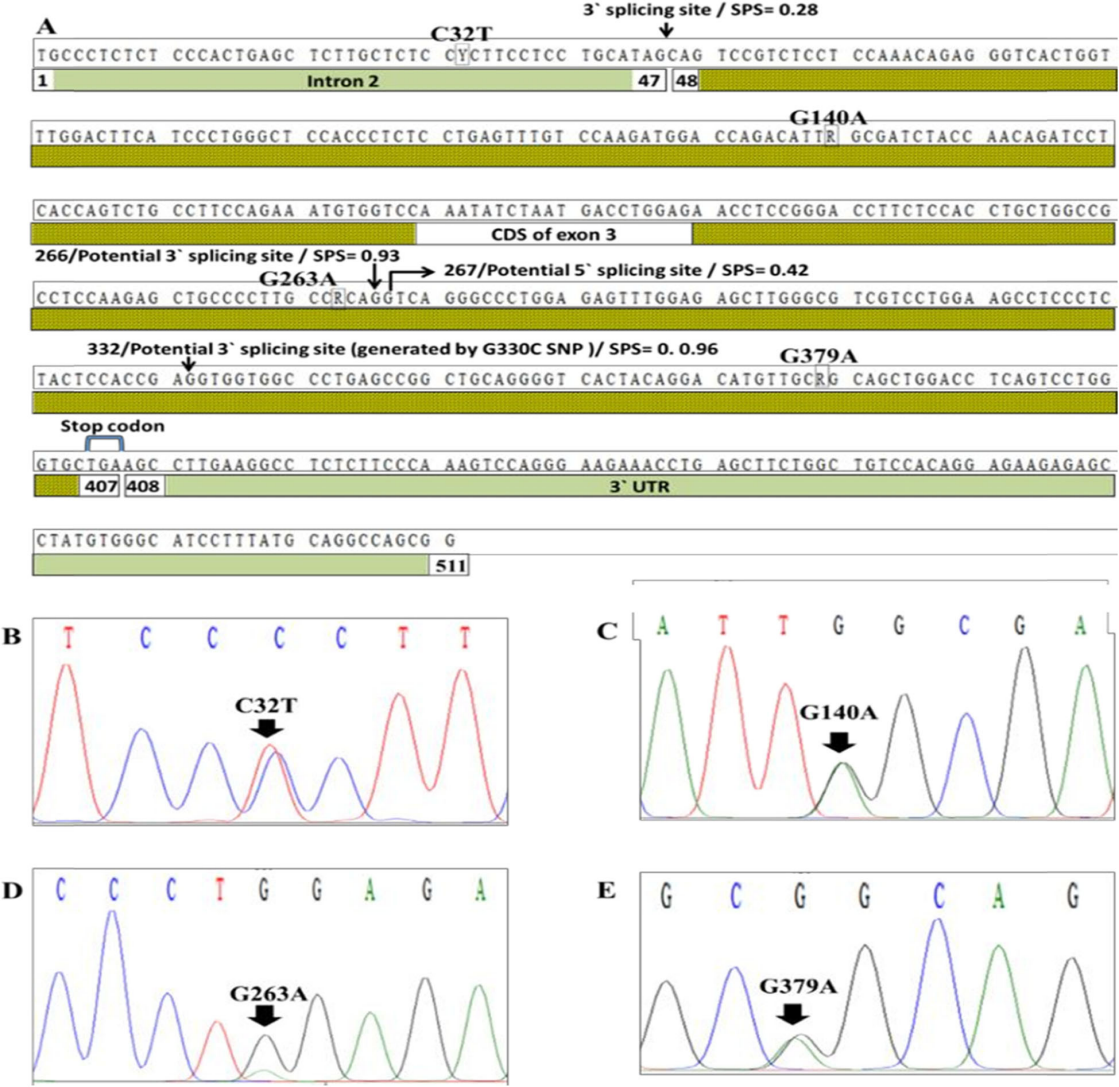
**a**–**e** The nucleotide sequence and the detected SNPs in the Egyptian river buffalo LEP gene amplified fragment. SPS: SplicePort score

Sequencing of the amplified region of the Egyptian buffalo LEP gene (GenBank accession numbers MF490262-MF490277) produced a nucleotide sequence of 511 bp which covers a part of intron 2 (1–47 bp), coding region of exon 3 (48–404 bp), stop codon (405–407 bp), and 3′ UTR (408–511 bp; Fig. 2a).

Within the Egyptian buffalo samples, 4 transition SNPs (C32T, G140A, G263A, and G379A) were revealed in Fig. 2. The alleles of the last 3 SNPs in addition to 2 transversion SNPs and 19 transition SNPs were found between sequenced amplicon and the other buffalo breed records in GenBank; 22 of these SNPs were exonic and 2 were intronic (Table 1).

**Table 1:** Detected SNPs in the amplified LEP sequence and its homologs *Bubalus bubalis* sequences in the GenBank database and previous studies

Region	SNP	Alleles	GenBank accession number or reference
Intron 2	T27C	C	KP897166.1, EU825672.1, GQ385228.1
Intron 2	C32T	C	HE605297.1, AH013754.2, AF387814.1, EU888289.1, JN689387.1, AY338973.2, KP897165.1, KP897166.1, DQ831143.1, DQ831142.1, EU825674.1, EU825673.1, EU825672.1, GQ385228.1, DQ490986.1
Intron 2	C33T	T	GQ385228.1
Exon 3	A114G	G	EU888289.1
Exon 3	G140A	G and A	[8]
		G	HE605297.1, AH013754.2, AF387814.1, EU194869.1, EU199796.1, EU888289.1, KP864439.1, NM_001290901.1, KP864440.1, KP864438.1, KP864436.1, KP864437.1, AY427959.1, JN689387.1,DQ676889.1, AY338973.2, AY177609.1, JQ045625.1, KP897165.1, KP897166.1, DQ831143.1, DQ831142.1, EU825674.1, EU825673.1, EU825672.1, GQ385228.1, DQ490986.1
		A	DQ676890.1
Exon 3	C163A	A	EU825672.1, EU825673.1, EU825674.1, GQ385228.1
Exon 3	T197C	C	AY177609.1, JQ045625.1
Exon 3	G206A	A	AY177609.1
Exon 3	A211G	G	JQ045625.1
Exon 3	C242T	T	EU078405.1
Exon 3	G263A	G and A	[8, 9]
		G	HE605297.1, AF387814.1, EU194869.1, EU199796.1, KP864439.1, NM_001290901.1, KP864438.1, AY427959.1, JN689387.1, DQ676890.1, DQ676889.1, AY177609.1, JQ045625.1, KP897165.1, KP897166.1
		A	AH013754.2, EU888289.1, KP864436.1, KP864437.1, KP864440.1, AY338973.2
Exon 3	G278A	G and A	AF387814.1
		A	KP864438.1, AY427959.1
Exon 3	G278A	G and A	[8]
Exon 3	T284C	C	AY177609.1, JQ045625.1
Exon 3	G288A	A	AY177609.1
Exon 3	C302T	T	AY177609.1, JQ045625.1
Exon 3	A310G	G	KP864436.1, KP864437.1, KP864438.1. KP864439.1, KP864440.1
Exon 3	C314T	T	AY177609.1, JQ045625.1
Exon 3	A322G	G	JN689387.1
Exon 3	G330C	C	JQ045625.1
Exon 3	C348T	T	JQ045625.1
Exon 3	T360C	C	HE605297.1
Exon 3	G379A	G and A	[8, 9]
		G	HE605297.1, AH013754.2, AF387814.1, EU199796.1, KP864439.1, NM_001290901.1, KP864440.1, KP864438.1, KP864436.1, KP864437.1, AY427959.1, DQ676889.1
		A	EU194869.1, DQ676890.1
Exon 3	G386A	A	KP864437.1
Exon 3	T398C	C	NM_001290901.1, AY427959.1

### Comparison of the amplified region in LEP gene among some species

**Fig. 3:**
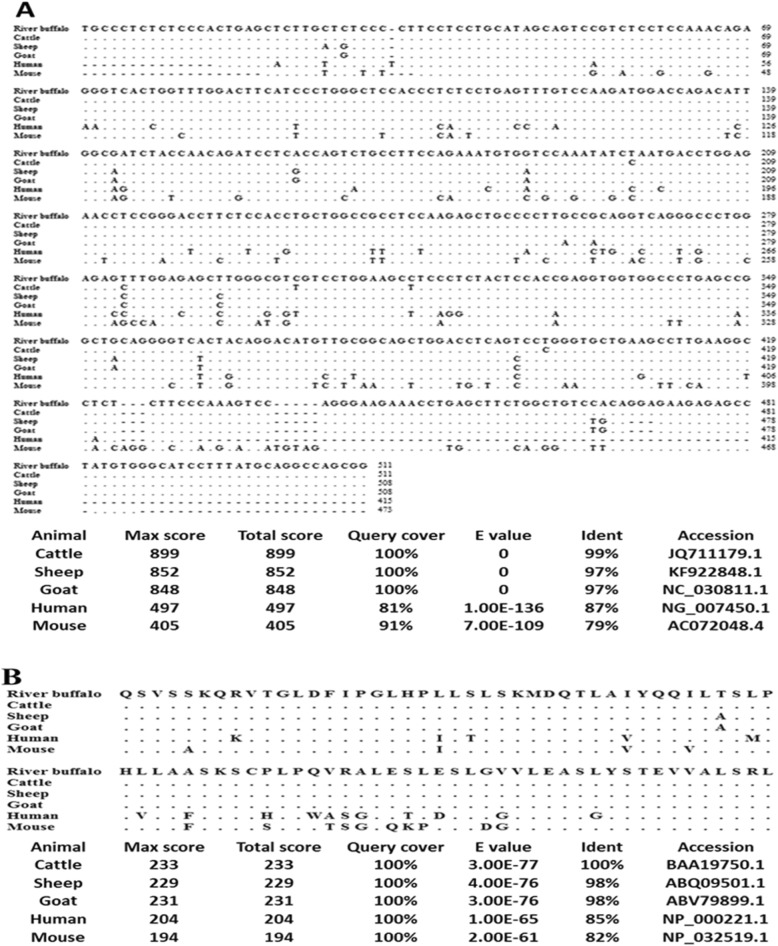
The nucleotides and translated amino acid sequences homology between the amplified region of LEP gene in Egyptian river buffalo and some species. Multiple sequence alignment of the nucleotides (**a**) and translated amino acids (**b**) sequences

The sequence of amplified LEP gene fragments and the translated amino acid sequence of the coding region within this fragment were aligned with the orthologous genes in human, mouse, sheep, cattle, and goat (Fig. 3).

### Predicting the effect of the detected SNPs within LEP gene on the splicing sites strength cis-acting splicing regulatory elements

SplicePort software was used to detect the strength of both known splicing sites (3′ splicing site of intron 2, 5′ splicing site of intron 3) and potential splicing sites within the amplified LEP gene fragment and the effect of the detected SNPs on them (Fig. 2a). Furthermore, using RegRNA 2.0 software, six SNPs (T27C, C32T, A114G, G263A, A310G, and G379A) were found to have several potential effects on RNA cis-regulatory elements (Table 2).

**Table 2:** The effect of detected SNP within buffalo LEP gene on the RNA cis-acting splicing regulatory elements in LEP hrRNA

SNP	Effect	Motif	Binding factor
T27C	Disrupts: intron silencer of c-src, exon n1 - *Mus musculus*	26 c**T**ctc 30	PTB/nPTB, KSRP, FBP, hnRNP H, hnRNP F
C32T	Creates: intron silencer of caspase-2, exon 9 - *Mus musculus*	32 **T**cttcc 37	PTB
A114G	Disrupts: exon enhancer of brca1, exon18 - *Homo sapiens*	111 ctg**A**gtt 117	SFRS1 (SF2/ASF)
G263A	Disrupts: asf/sf2 - exonic splicing enhancer	262 c**G**caggt 268	ASF/SF2
	Creates: SRp40-exonic splicing enhancer - *Homo sapiens*	263 **A**cagg 267	SRp40
	Creates: SRp55 - exonic splicing enhancer - *Homo sapiens*	261 cc**A**cagg 267	SRp55
A310G	Disrupts: GH exon 5 exon splicing enhancer - *Bostaurus*	308 gg**A**ag 312	ASF/SF2
G379A	Disrupts: SRp55-exonic splicing enhancer - *Homo sapiens*	376 tgc**G**gc 381	SRp55
	Creates: SRp40-exonic splicing enhancer - *Homo sapiens*	376 tgc**A**gc 381	SRp40

### Effect of the detected non-synonymous SNPs in LEP gene

**Fig. 4:**
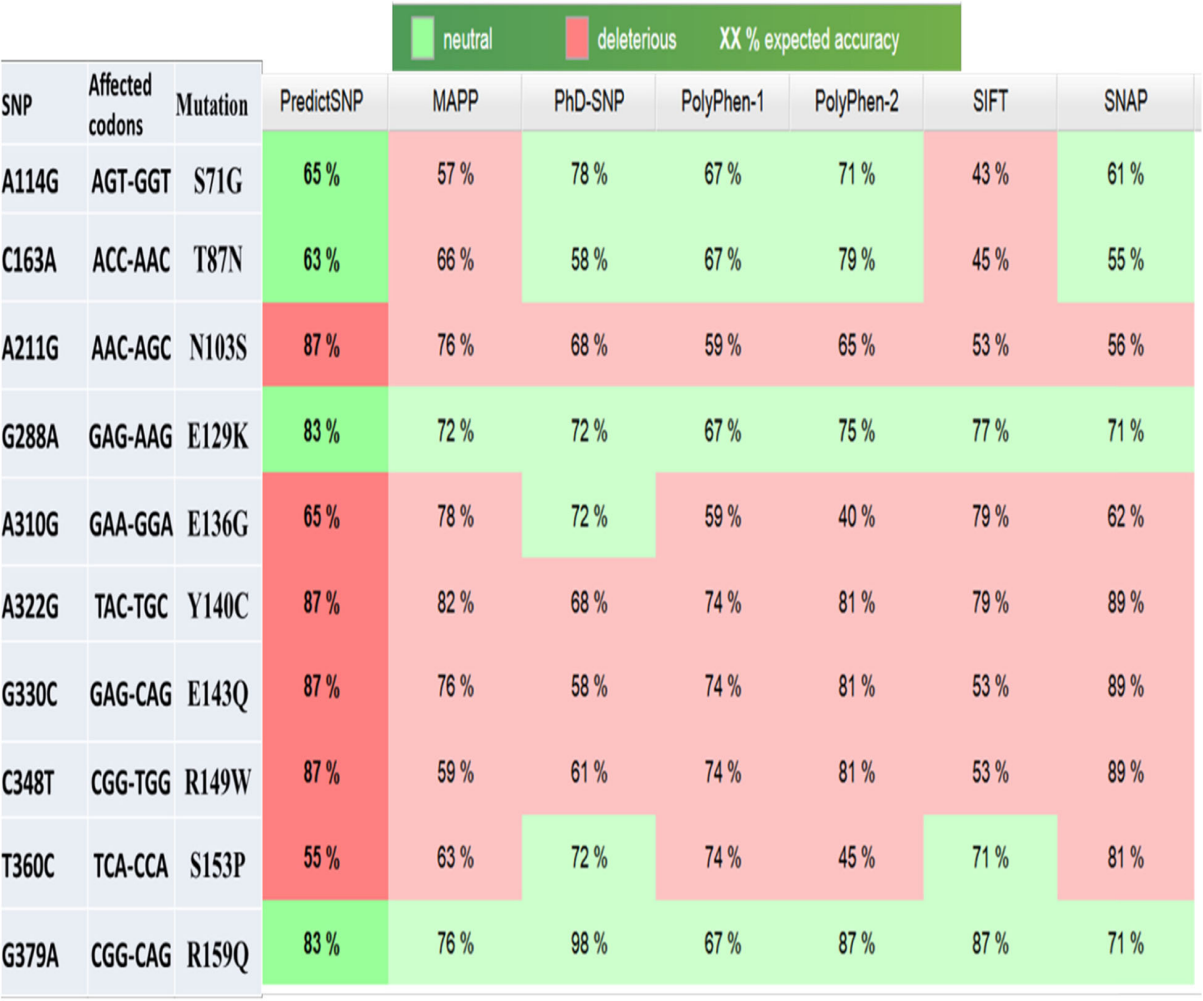
The effect of non*-*synonymous SNPs on river buffalo LEP polypeptide

**Fig. 5:**
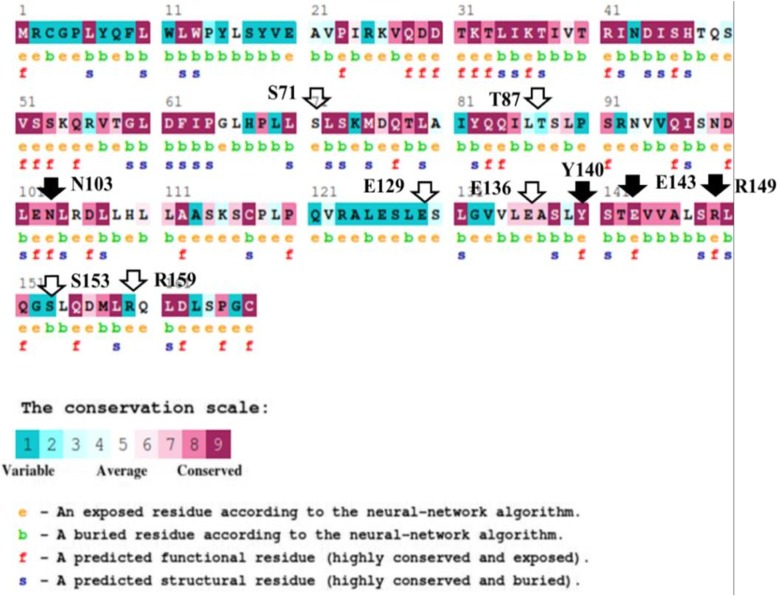
The conservation degree of the leptin polypeptide amino acids. The mutated amino acids were marked by solid arrows (conserved sites) or empty arrows (non-conserved sites)

A114G, C163A, A211G, G288A, A310G, A322G, G330C, C348T, T360C, and G379A SNPs were found to be non-synonymous which caused S71G, T87N, N103S, E129K, E136G, Y140C, E143Q, R149W, S153P, and R159Q mutations in LEP polypeptide, respectively (based on the CDS of LEP gene in the GenBank record NW_005782251.1). The non-synonymous SNPs in LEP gene were evaluated by PredictSNP which combined the results of six programs which utilize different methods to predict the deleterious effect of the non-synonymous SNP (Fig. 4). The amino acid conservation analysis on 180 leptin amino acid sequences from many species was performed using ConSurf server (Fig. 5), and the amino acids were classified based on conservation score ranging from 1 (variable) to 9 (conserved).

**Fig. 6:**
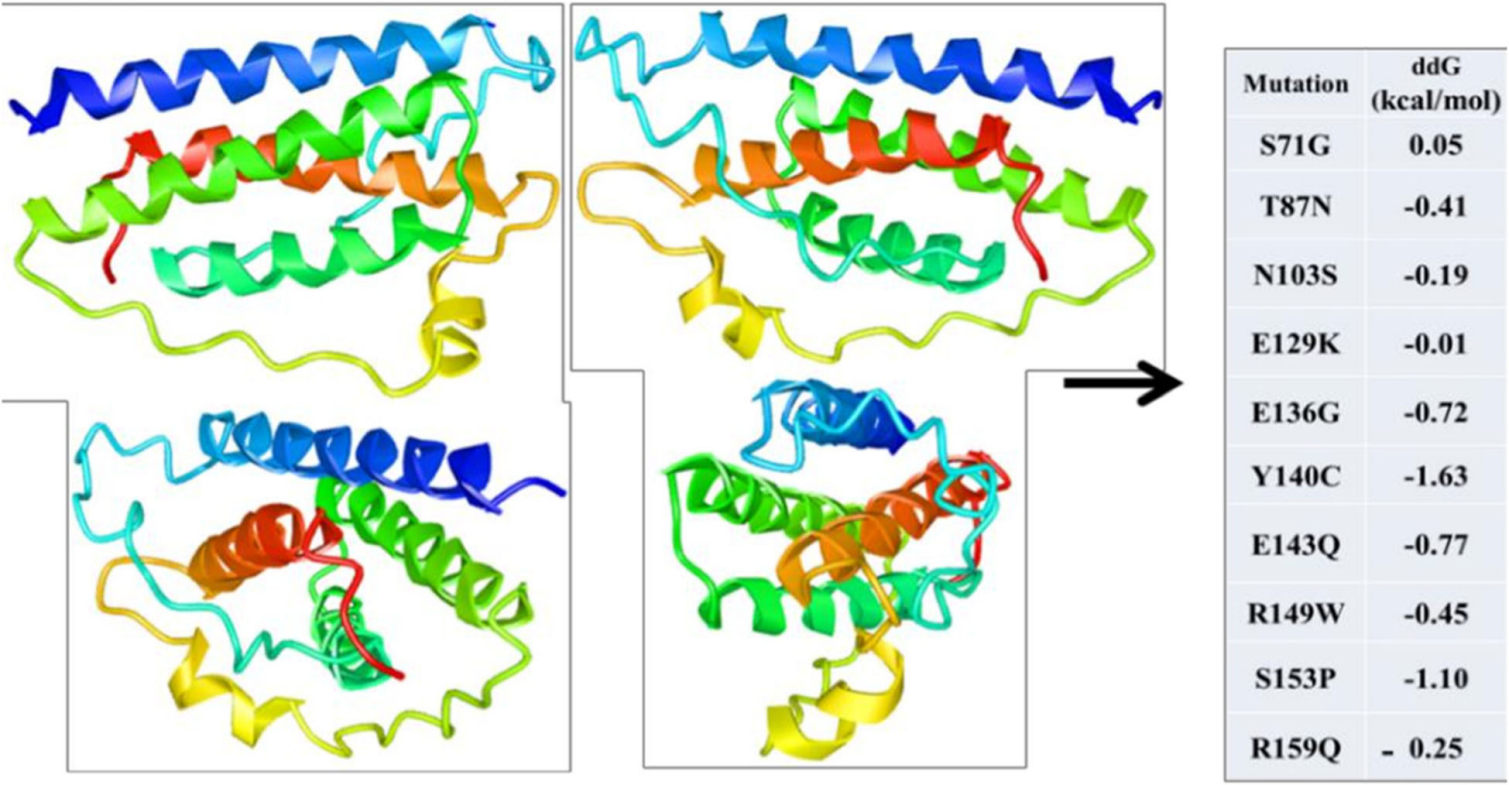
The predicted 3D tertiary structure of the mature river buffalo leptin peptide from several views and the effect of the detected mutations on its stability

The 3D tertiary structure of river buffalo mature leptin peptide (146 amino acids) was predicted using the I-TASSER server, and the effect of the discovered non-synonymous on the stability of tertiary structure of mature leptin peptide was predicted using the INPS3D server based on free energy difference (ΔΔG = the free energy change from wild type to mutant) which was calculated (Fig. 6).

## Discussion

The present investigation studied the polymorphism in LEP gene which related to fertility in 81 female Egyptian river buffaloes. PCR-RFLP pattern using *Eco91I* of the target fragment showed that all the investigated buffaloes had CC genotype which was confirmed by direct sequencing. In agreement with the current results, some investigators confirmed the CC genotype as the only detected genotype among several river buffalo breeds [3, 8–12]. But in cattle, the frequencies of CT and TT genotypes were 34% and 8% (Liefers et al. [4]), 47.03% and 9.13% [7], 38.8% and 2.4% [5], and 33% and 6% [6], respectively. Some studies found that the SNPs which are related to some reproduction traits in cattle were monomorphic in Egyptian buffaloes [23, 24].

Interestingly, both *Eco91I* [7] and *HphI* [4] enzymes which are used for C to T SNP (A59V mutation) detection in cattle are not suitable in any future studies on Egyptian buffalo to detect this A59V mutation because a G140A SNP was discovered by sequencing of LEP amplicon (Fig. 1c), and the A allele of the discovered SNP disrupts *Eco91I* and *HphI* restriction sites. This finding means that *Eco91I* and *HphI* will not be able to differentiate the 2 alleles of A59V mutation. It could be suggested that the 2 alleles of LEP gene C to TSNP (A59V) could be differentiated in any future studies on river buffalo using a PCR reaction which could be carried out by the same forward primer designed in this study in combination with the mutated primers (5′ CTGGTGAGGATCTGTTGGT**C**GATC 3′) or (5′CTGGTGAGGATCTGTTGGT**T**GATC 3′) which will produce an amplicon that has the restriction site of *PvuI* (CGATCG which will cut C allele) or *BclI* (TGATCA which will cut T allele), respectively.

Four SNPs (C32T, G140A, G263A, and G379A) were detected within the Egyptian buffalo samples. The alleles of G140A, G263A, and G379A SNPs in addition to 21 SNPs were found between in the leptin gene in the sequenced fragment of the Egyptian buffalo and the other buffalo breed records for the same gene in GenBank. The T allele of C32T intronic SNP was unique and was detected in Egyptian buffalo within this investigation only and was not detected in any buffalo records in GenBank or previous studies on river buffalo [3, 8–12].

The Brazilian buffalos in the study of Vallinoto et al. [3] had both alleles of G263A and G278A SNPs but had the G allele only of G140A and G379A SNPs compared to Egyptian buffaloes, and the Brazilian and Egyptian breeds are monomorphic for G386A SNP. Both of the Italian [8] and Egyptian buffaloes were polymorphic for G140A and G379A SNPs, but in contrast to Egyptian buffaloes, the Italian buffaloes were monomorphic for G263A SNP and polymorphic for G278A SNP. The Philippine buffaloes [9] had the two alleles of G263A and G379A SNPs exactly like the Egyptian breeds.

Compared to the Egyptian buffaloes, the Indian buffaloes [10] were polymorphic for G379A SNP and also were monomorphic for G140A and G263A SNPS (had G alleles of both SNPs), and the Egyptian buffaloes had the C allele of C242T SNP while the Indian buffaloes had T allele. In the studied sample of Italian buffaloes [12], the animals had G alleles of G140A, G263A, and G379A SNPs in addition to C allele of T360C SNP in opposition to Egyptian buffaloes.

Blasting of the nucleotide sequence of Egyptian river buffalo LEP gene amplified region against GenBank database displayed that cattle had the highest homology score (99%) compared to sheep (97%), goat (97%), human (87%), and mouse (79%). On the other hand, the homology of the translated amino acid sequence from full coding region of Egyptian river buffalo LEP gene-exon 3 and the similar sequence in cattle, sheep, goat, human, and mouse was 100%, 98%, 98%, 85%, and 82%, respectively. The homology of the translated amino acid sequence was higher in the target organisms than their DNA sequences homology.

Using the SplicePort software, the score of the 3′ splicing site in the end of intron 2 (in the position 47) was calculated to be 0.28 which was lower than the score of the intron 2–3′ splicing site in human (0.31) which could be replaced by another 3′ splicing site located 3 bp downstream it that lead to protein isoform which lacks glutamine at position 49 of the mature peptide [25]. This weak splicing site could be affected by flanked cis-acting splicing regulatory elements [26]. The SplicePort software detects potential 3′ splicing sites with a higher score than the regular 3′ splicing site located in the positions 266 (0.93) and 469 (0.87) in addition to 5′ splicing site (0.42) in the positions 267. G330C SNP generated a 3′ splicing site in position 332 with a score equals 0.96. These sites may act as cryptic splicing sites which could be activated by some mutations or naturally without any mutations leading to change in the final transcription products [27–31].

Moreover, six SNPs were predicted to have different effects on RNA cis-regulatory elements. Table 2 shows that T27C and C32T SNPs are very close to the 3′ splicing site of intron 2 and the first SNP disrupts an intron splicing silencer while the lastone creates a new intron splicing silencer. In the exon 3, both A114G and A310G SNPs disrupt two exonic splicing enhancers, but G263A and G379A SNPs disrupt two exonic splicing enhancers and in the same time the two SNPs create new exonic splicing enhancers. Disruption, creation, or changing the number of the cis-acting splicing regulatory elements could change the splicing efficiency and affect the different consequence processes like alternative splicing and intron retention which affect the final protein sequence and structure [32, 33].

Ten non-synonymous were found among the detected SNPs, and their potential effects on LEP protein functions were predicted (Fig. 4). The results showed that all the programs classified E129K and R159Q mutations as neutral mutations with a total PredictSNP expected accuracy of 83%. S71G and T87N mutation were classified as neutral mutations by PHD-SNP, POLYPHEN-1, POLYPHEN-2, and SNAP and as deleterious mutations by MAPP and SIFT, so the PredictSNP software classified them as neutral mutations but with 65 and 63% accuracy only. Furthermore, N103S, Y140C, E143Q, and R149W were evaluated by the six software as deleterious mutations with 87% combined accuracy by PredictSNP. E136G and S153P mutations were predicted as deleterious mutation by all the programs except PHD-SNP in the case of E136G and PHD-SNP and SIFT in the case of S153 with a 65 and 55% PredictSNP accuracy, respectively.

The amino acid conservation analysis showed that the amino acids S71, T87, E129, E136, S153, and R159 are not conserved in LEP polypeptide. The amino acids Y140 and E143 had the highest conservation score followed by the amino acids N103 and R149. These findings confirm that the conserved amino acids had an important functional or structural role on the native LEP protein [22], so the mutation in these amino acids could have a damaging effect on LEP protein.

Finally, the effect of revealed non-synonymous on the stability of 3D tertiary structure of river buffalo mature leptin peptide was predicted (Fig. 6). S71G only increased the stability of the leptin protein by 0.05 kcal/mol. T87N, N103S, E129K, E136G, Y140C, E143Q, R149W, S153P, and R159Q lowered the stability of mature leptin peptide tertiary structure by − 0.41, − 0.19, − 0.01, − 0.72, − 1.63, − 0.77, − 0.45, − 1.10, and − 0.25 kcal/mol, respectively. Y140C and S153P mutations reduced the stability of mature leptin peptide tertiary structure by more than − 1 kcal/mol which added more evidence supporting the damaging effect of these mutations [34]. Finally, the amino acid N103 in the leptin polypeptide is conserved in human and river buffalo, and the N to K mutation which occurs in this amino acid has a damaging effect on the physiological function of human LEP protein [35].

## Conclusions

In this study, the polymorphism in LEP gene in 81 female Egyptian river buffalo was investigated by PCR-RFLP Eco91I, and all the animals had the CC monomorphic pattern. Four SNPs were revealed among the tested animals by sequencing a 511-bp fragment from LEP gene. Moreover, 21 SNPs were found between the sequenced amplicon and the homologous buffalo records in Genbank. The homology between the amplified LEP gene fragment in buffalo and cattle, sheep, goat, human, and mouse on the nucleotides sequence level was 99, 97, 97, 87, and 79%, respectively, and on the translated amino acids sequence level was 100, 98, 98, 85, and 82%, respectively. The T27C SNP disrupts an intronic splicing silencer. The A114G, A310G, G263A, and G379A SNPs disrupt exonic splicing enhancers, and the last 2 SNPs create new exonic splicing enhancers. The A114G, C163A, A211G, G288A, A310G, A322G, G330C, C348T, T360C, and G379A SNPs cause S71G, T87N, N103S, E129K, E136G, Y140C, E143Q, R149W, S153P, and R159Q amino acid mutations. N103S, E129K, E136G, Y140C, E143Q, and S153P were classified as deleterious mutations. Y140, E143, N103, and R149 were the most conserved among the mutated amino acids. S71G only increased the stability of the leptin protein while the remaining mutations decreased it.

## Data Availability

All data generated or analyzed during this study are included in this published article.

## Abbreviations

LEP: Leptin
MAS: Marker-assisted selection
PCR: Polymerase chain reaction
RFLP: Restriction fragment length polymorphism
SNP: Single nucleotide polymorphism
SPS: SplicePort score
SSCP: Single-stranded conformation polymorphism
